# Role of Endocytosis Pathways in Electropermeablization of MCF7 Cells Using Low Voltage and High Frequency Electrochemotherapy

**DOI:** 10.22074/cellj.2021.7203

**Published:** 2021-08-29

**Authors:** Sajedeh Yadegari-Dehkordi, Seyed Mohammad Firoozabadi, Mehdi Forouzandeh Moghadam, Zeinab Shankayi

**Affiliations:** 1. Department of Medical Physics, Faculty of Medical Sciences, Tarbiat Modares University, Tehran, Iran; 2. Department of Medical Biotechnology, Faculty of Medical Sciences, Tarbiat Modares University, Tehran, Iran

**Keywords:** Bleomycin, Electrochemotherapy, Endocytosis, Low Intensity Electric Field

## Abstract

**Objective:**

The cell membrane is a major barrier for delivery of hydrophilic drugs and molecules into the cells. Although low voltage and high frequency electric fields (LVHF) are proposed to overcome the cell membrane barrier, the mechanism of membrane permeabilization is unclear. The aim of study is to investigate endocytosis pathways as a possible mechanism for enhancing uptake of bleomycin by LVHF.

**Materials and Methods:**

In this experimental study, MCF-7 cells were exposed to bleomycin or to electric fields with various strengths (10-80 V/cm), frequency of 5 kHz, 4000 electric pulse and 100 µs duration in the presence and absence of three endocytosis inhibitors-chlorpromazine (Cpz), amiloride (Amilo) and genistein (Geni). We determined the efficiency of these chemotherapeutic agents in each group.

**Results:**

LVHF, depending on the intensity, induced different endocytosis pathways. Electric field strengths of 10 and 20 V/cm stimulated the macropinocytosis route. Clathrin-mediated endocytosis was observed at electric field intensities of 10, 30, 60 and 70 V/cm, whereas induction of caveolae-mediated endocytosis was observed only at the lowest electric field intensity (10 V/cm).

**Conclusion:**

The results of this study imply that LVHF can induce different endocytosis pathways in MCF-7 cells, which leads to an increase in bleomycin uptake.

## Introduction

Although the cell membrane is a major barrier for intracellular delivery of polar molecules and ions, it does not allow drugs, DNA and proteins to enter the cell because of their sizes and/or charges. Therefore, targeted and controlled drug and gene delivery into cells and tissues remains a major research challenge. Physical and non-physical methods have been developed to overcome this challenge. Electroporation, a well-known physical technique, is an electrical method that transfers non-permeable drugs or other molecules across the plasma cell membrane by pulse electric fields. When the cells are exposed to high amplitude and short duration electric pulses, orientation of membrane phospholipids change and hydrophilic pores are formed in the cell membrane, which enables molecules to pass through the membrane (1). Electroporation can be used to deliver small impermeable molecules or exogenous macromolecules through the membrane into the cell (2). An example of delivery of small molecules is electrochemotherapy (ECT), which is the combination of electroporation with chemotherapeutic drugs. Another example of delivery of macromolecules is DNA electro-transfection or electrogentherapy.

The amplitudes of external electric field should reach a critical value (on the order of hundreds to thousands of volts per centimeter) to induce electroporation in a lipid bilayer. Accordingly, researchers extensively focused on high voltage electrical fields for both ECT and electrogentherapy. However, the results of studies show that low voltage electric fields can increase cell permeability for both drugs (small molecules) and proteins (macromolecules). Antov et al. demonstrated that low pulse electric fields in the range of 2.5-20 V/cm enhanced the uptake of the macromolecules such as dextran conjugated to fluorescein-5- isothiocyanate (dextran-FITC, 2000 kDa) and bovine serum albumin (BSA) conjugated to FITC (66 kDa) into the cells (3). The reason for this electropermeablization was explained by endocytosis induction. The results of another study showed that electric pulses (20-250 V/cm) could improve the efficiency of chemotherapy drugs (4, 5). Shankayi et al. (6-8) conducted several studies and demonstrated that low intensity (50 to 150 V/cm) and high frequency (4-6 KHz) with 4000 pulses of electric fields increased the uptake of small bleomycin molecules (<1.5 kDa), a cell-impermeable chemotherapy agent, into the cell cytoplasm. They found that low voltage and high frequency electric fields (LVHF) increased cell permeability more effectively than standard ECT with eight pulses, 1000 V/cm and 1 Hz frequency (8). However, increasing cell permeability by LVHF is not yet understood. 

Therefore, in the present study, we sought to determine the mechanism of LVHF to enhance cell permeability. The amplitude of this low voltage electric field is lower by several orders of magnitude than the electroporation threshold; thus, the formation of pores in the cell membrane is vague and other mechanisms should be considered. According to the results of the Antov et al. (3) study that was based on the uptake of macromolecules through the endocytosis pathway by a low voltage electric field, we assumed that endocytosis was a mechanism for increasing the efficiency of chemotherapy (small molecules) by LVHF. Endocytosis is a cellular process by which a cell takes up extracellular macromolecules into the cell cytoplasm. Many endocytic pathways exist within the cell to facilitate intracellular uptake of external macromolecules and to shuffle them from the plasma membrane to destinations throughout the cell. Several molecular mechanisms have been described for endocytosis: clathrin-dependent, caveolae-mediated, macropinocytosis, and clathrin- and caveolae-independent endocytosis. The use of pharmacological inhibitors is a well-known approach for studying the role of the endocytic pathway in the delivery of various substances such as nanoparticles, gene carriers and biomarkers (9-12). A variety of pharmacological inhibitors of endocytosis have been developed and each could transiently block specific pathways of endocytosis. In the present study we used three endocytosis inhibitors-chlorpromazine (Cpz), genistein (Geni) and amiloride (Amilo). Cpz blocks clathrin-mediated endocytosis (13, 14). Amilo is used as an inhibitor of macropinocytosis and Geni, a tyrosine kinase inhibitor, prevents the depolarization of actin by precluding the caveolae-mediated endocytosis pathway (15, 16). 

Here, we investigated the role of endocytosis pathways for enhancing bleomycin uptake by LVHF (10-80 V/ cm). We also investigated the dependence of the clathrin-mediated endocytosis, caveolae-mediated endocytosis and macropynocytosis pathways on the intensity of the electric field. Bleomycin is a chemotherapy drug which, due to its polar property, is unable to permeate the cell. However, the entry of a small number of bleomycin molecules into the cell can induce high toxicity and mitotic cell death. Thus, it is considered as a sensitive marker of cellular uptake. In present study, we used three endocytosis inhibitors because each transiently blocks specific pathways of endocytosis to investigate their effects on ECT with LVHF (10-80 V/cm) in MCF-7 cells.

## Materials and Methods

### Cell culture

The Ethics Committee of Tarbiat Modares University, Tehran, Iran approved this experimental research study (IR.TMU.REC.1395.492). MCF-7 cells were grown in RPMI-1640 medium (Invitrogen, Gibco, USA) that contained 10% foetal bovine serum (FBS, Invitrogen, Gibco, USA) and 1% antibiotics (penicillin 50 units/ml-streptomycin 50 mg/ml). Incubation was carried in 5% CO_2_ at 37˚C. For the assay, the cells were centrifuged for 5 minutes at 1000 rpm and resuspended in RPMI. The cell suspension was prepared at a concentration 1×10^6^ cells/ml. 

### Electric field exposure

Generation and application of electric pulses to the cells was performed with an ECT-SBDC that was designed and made in the Small Business Development Centre and Electromagnetic Laboratory of the Medical Physics Department of Tarbiat Modares University (Tehran, Iran). A sample that contained 30000 cells was placed between two gold electrodes (parallel plate electrodes with an inter electrode distance of 10 mm). The pulse number, frequency and duration of pulses were the same in all the experiments and the voltage of the electric field was variable. The cells were exposed to a 4000 electric pulse with a 100 µs duration and frequency of 5 kHz. The electric pulses had an amplitude of 10-80 V/cm with a step of 10 V/cm and total exposure time of 800 ms (6, 7).

### Bleomycin (CT, chemotherapy drug)

Bleomycin is a non-permeable chemotherapy drug. In the ECT groups, we added 1 μM bleomycin (Nippon Kayaku Co. Ltd., Japan) to the cells one minute before pulsing. 

### Determination of induced endocytosis

We separately examined the effects of three endocytosis inhibitors on ECT efficiency to specify induced endocytosis. The endocytosis inhibitors were Cpz (10 µM), Geni (200 µM) and Amilo (400 µM) (Sigma Aldrich, St Louis, MO, USA) (17-19). Cpz inhibits clathrin-mediated endocytosis (13, 14). Geni affects raft/caveolae-mediated endocytosis and Amilo alters micropinocytosis (15, 16).

First, after addition of the inhibitor, the cells were incubated at 37˚C for one hour. Subsequently, we added bleomycin to the ECT group or medium to the electric group to the cells and the electric fields were applied using the above mentioned protocol. The cells were then seeded in a 96-well plate (10000 cells per well) and placed in an incubator at 37˚C and 5% CO_2_ . After 48 hours, cell viability was determined by the [3-(4,5-dimethylthiazol-2-yl)-2,5-diphenyltetrazolium bromide] (MTT) assay.

The relative synergy is a parameter that shows the efficiency of combined treatments. Relative synergy for ECT, which is the combination of bleomycin (CT) and electric (E) field, was calculated as follows:

$$\mathrm{Relative synergy factor}=\frac{\mathrm{percentage of cell deaths induced by ECT}}{\mathrm{percentage of cell deaths induced by E+ percentage of cell deaths induced by CT}}$$

A relative synergy greater than one implies additional effects compared with the sum of the individual treatment factors (20).

### [3-(4,5-dimethylthiazol-2-yl)-2,5-diphenyltetrazolium bromide] (MTT) assay

The MTT assay was used to determine cell viability. When bleomycin enters a cell, it causes cell death. Therefore, the cell viability assay can indicate cell permeability. The MTT (Invitrogen, Gibco, USA) assay was conducted as follows: 20 μl MTT solution [5 mg MTT/ml in phosphate-buffered solution (PBS)] was added to wells and after a four hour incubation period, dimethyl sulfoxide (DMSO) was added to the wells and the solution was mixed. Optical density was read by an optical Elisa Reader (BioTek,Cytation 5, USA) at a 570 nm wavelength filter (21).

### Statistical analysis

All results are presented in bar graphs where vertical bars represent the standard deviation of the mean. Statistical analyses were performed using SPSS Inc. (Copyright 1993-2007, Polar En-gineering and Consulting, Sept 13, 2007) for Windows 16.0. All data were tested for normality. Statistical data were compared by one-way analysis of variance (ANOVA) followed by the least significant difference (LSD) and independent t test. P<0.05 were considered significant for rejection of the null hypothesis. At least three independent experiments were performed on different days under the same conditions. There were three samples for each experimental group.

## Results

### Influence of inhibitors on cell viability, bleomycin uptake and electric field

**Fig.1:**
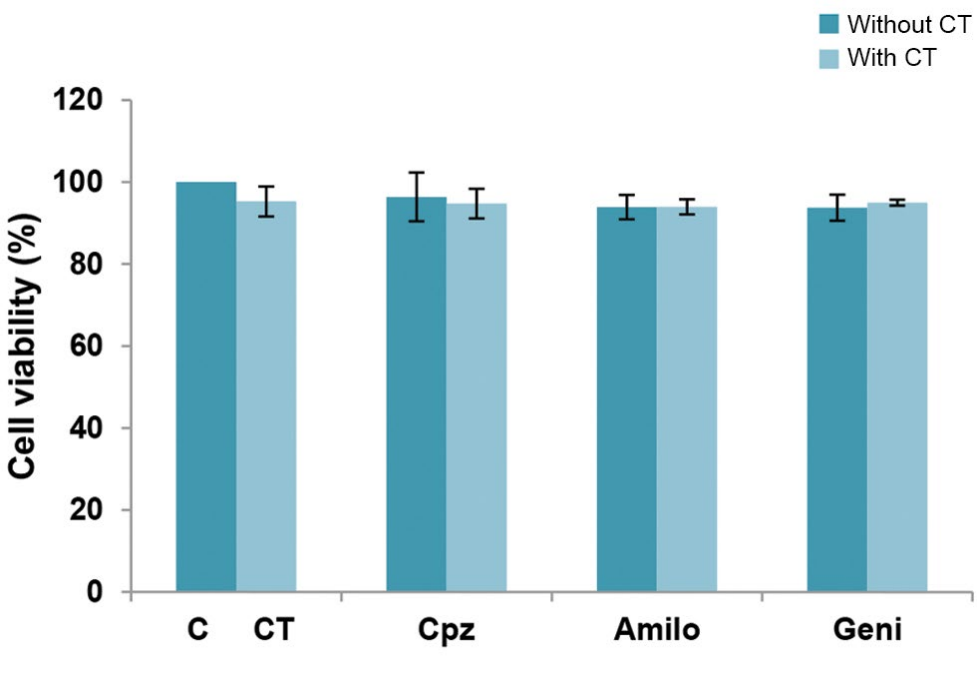
The effect of endocytosis inhibitors on cell viability and bleomycin (CT) uptake. The following concentrations of endocytosis inhibitors were used: 10 µM of chlorpromazine (Cpz), 400 µM of amiloride (Amilo) and 200 µM of genistein (Geni). The data are presented as mean cell viability ± standard deviation of the mean. P<0.05 indicates statistical significance.

Figure 1 shows the toxic effects of the endocytosis inhibitors on the MCF-7 cells. The endocytosis inhibitors (Cpz, Geni and Amilo) did not cause any significant decrease in cell viability compared to the control group. We observed a reduction in cell viability of 1.4% for Cpz, 7.7% for Geni and 2.7% for Amilo.

**Fig.2:**
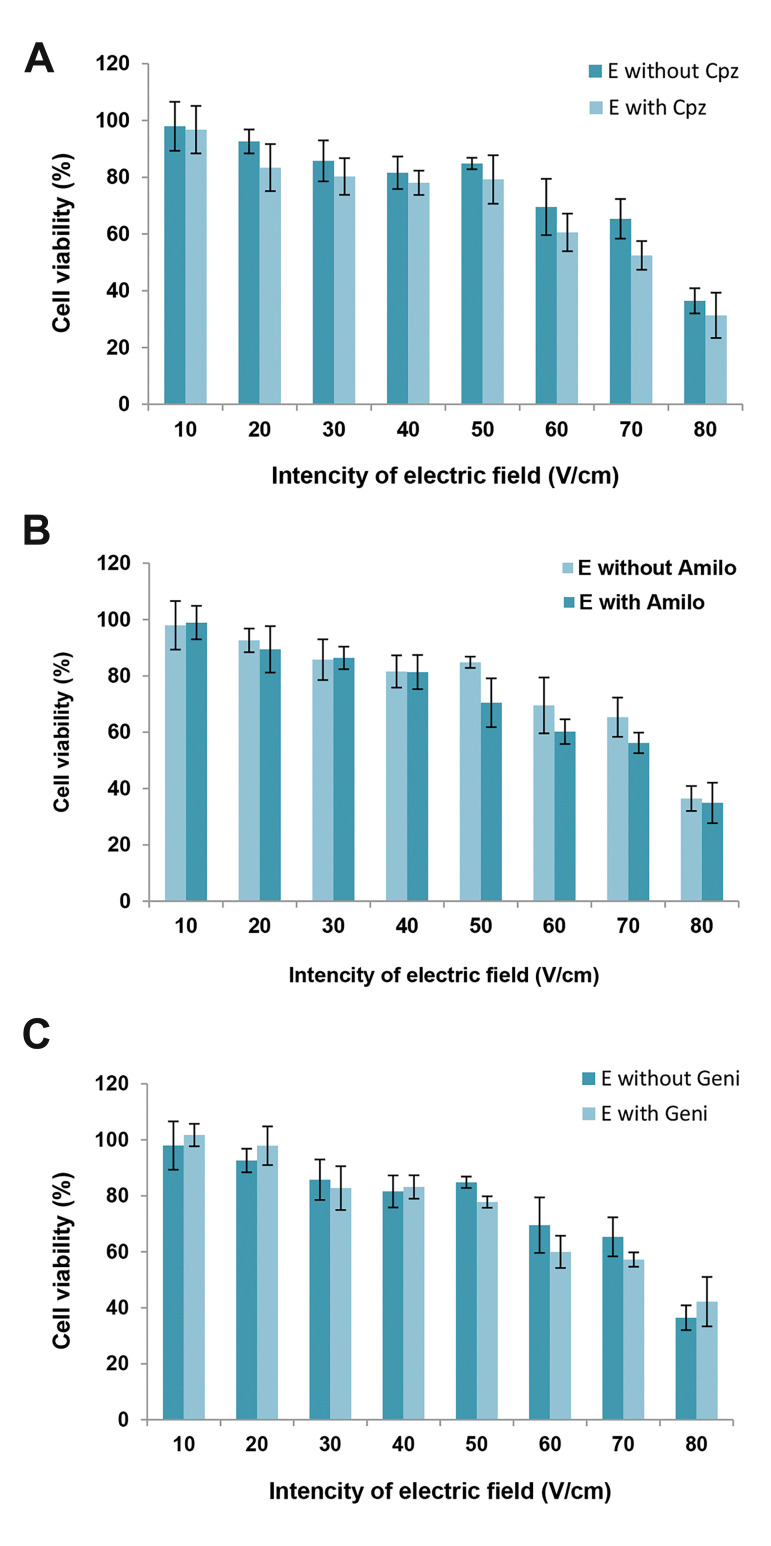
The toxic effects of chlorpromazine (Cpz), genistein (Geni), and amiloride (Amilo) on the electric (E) field treated cells. In Graphs (A) to (C), the cell viability was measured 48 hours after cell treatment with electric field in the absence and presence of **A.** 10 μM chlorpromazine (Cpz), **B. **400 μM amiloride (Amilo), and **C. **200 μM genistein (Geni). The data represent mean cell viability ± standard deviation of the mean. P<0.05 indicates statistical significance.

Since ECT is a combination of a chemotherapeutic agent (bleomycin) and an electric field, it is necessary to determine the effects of the inhibitors on each agent. According to the results, cell viability in the bleomycin (CT) with inhibitor groups did not appear to have any significant difference compared to the bleomycin group (Fig .1). Cell viability of the electric field-exposed cells in the presence of the inhibitors also did not differ from viability of electric field-exposed cells (Fig .2). Figures 1 and 2 show that the inhibitors did not have any effects on the toxicity of the bleomycin and electric field induction.

### The effect of inhibitors on electrochemotherapy

**Fig.3:**
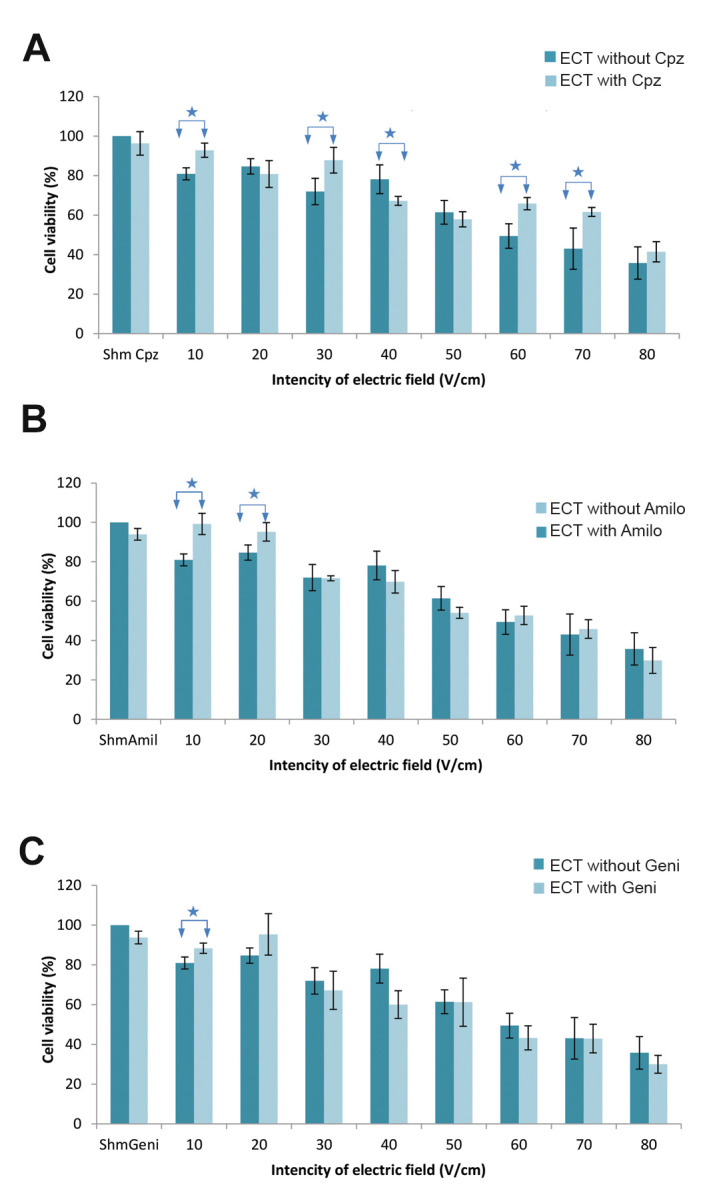
Electrochemotherapy (ECT) effect in the presence and absence of endocytosis inhibitors. In graphs A-C, we measured cell viability 48 hours after treatment with ECT in the absence and presence of: **A. **10 μM chlorpromazine (Cpz), **B. **400 μM amiloride (Amilo), and **C.** 200 μM genistein (Geni). The data represent the mean cell viability ± standard deviation of the mean. *; P<0.05 indicates statistical significance.

In this study, we investigated the effects of three endocytosis inhibitors - Cpz, Amilo and Geni on ECT efficiency. The cell viability of the ECT groups in the presence of these inhibitors were compared with ECT groups in the absence of the inhibitors. Figure 3A-C shows the results for each individual inhibitor.

ECT groups treated with Cpz had a significant increase in cell viability compared to the ECT groups without Cpz at the electrical intensities 10, 30, 60 and 70 V/cm (Fig .3A).

Figure 3B shows that Amilo significantly increased cell viability in the ECT groups with 10 and 20 V/cm intensities compared to the ECT group without Amilo.

There was a significant increase in cell viability of the ECT group in the presence of Geni only with the 10 V/cm electric field compared the ECT group without Geni (Fig .3C). 

### Electrochemotherapy

**Fig.4:**
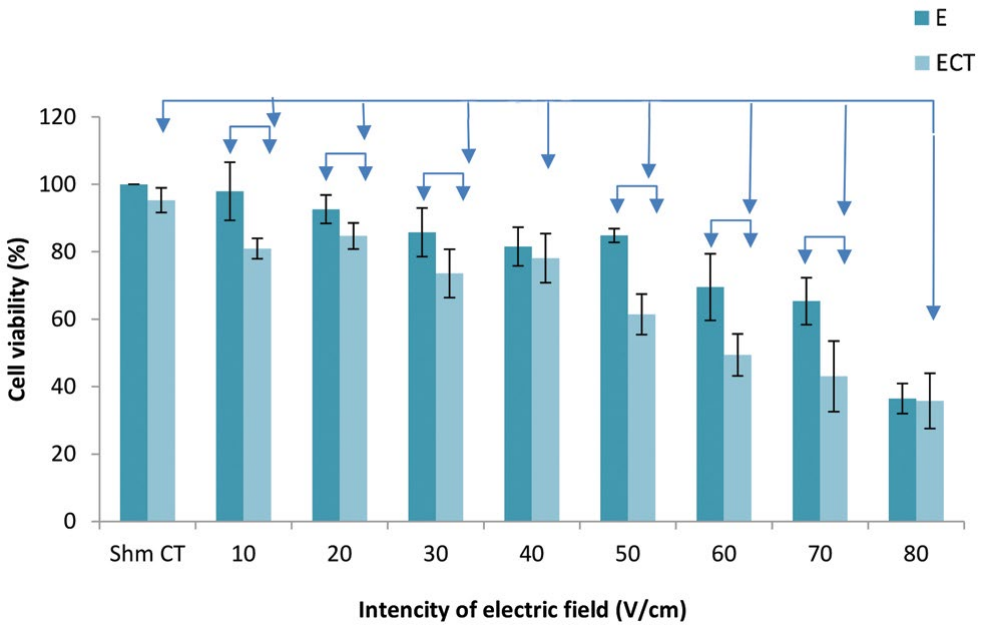
The effect of electrochemotherapy (ECT) compared to bleomycin (CT) and electrical pulse (E) alone. The data represent mean cell viability ± standard deviation of the mean. P<0.05 indicates statistical significance.

Figure 4 shows the ECT efficiency for the electric field intensities of 10-80 V/cm. The outcomes of ECT were compared with the effects of electric pulses alone and in the bleomycin groups. The ECT groups all showed significant reductions in cell viability compared to the bleomycin group. The results showed a significant difference in ECT efficiency compared with the electric group, with the exception of the 40 and 80 V/cm electric field intensities. 

Table 1 shows the relative synergy for the ECT treatment. Synergy was observed at all voltages, except for the 40 and 80 V/cm treatments. 

**Table 1:** Relative synergy for the electrochemotherapy (ECT) treatment


Voltage (V/cm)	10	20	30	40	50	60	70	80

Relative synergy	2.8	1.2	1.3	0.9	1.9	1.4	1.4	0.9


## Discussion

Bleomycin is a chemotherapy non-permanent and polar agent. The combination of bleomycin with electric fields (ECT) increased the toxicity of bleomycin. The synergistic effect for the ECT groups demonstrated that the LVHF electric field facilitated the entrance of bleomycin into the cell. One of the reasons for this synergy was endocytosis induction by the electric field, which increased the uptake of bleomycin. Our results showed that the presence of the inhibitor led to a reduction in bleomycin toxicity and an increase in cell viability for the ECT groups. Therefore, a possible route for intracellular delivery of bleomycin into a cell is via the endocytosis pathway.

We used three endocytosis inhibitors, Cpz, Amilo and Geni. Cpz inhibits the clathrin-mediated endocytosis pathway by blocking the function of AP2, one of the key adaptor proteins in clathrin-mediated endocytosis (13, 14). Geni is a tyrosine kinase inhibitor that prevents the depolarization of actin by precluding caveolae-mediated endocytosis (15). Amilo also prevents macropinocytosis by inhibiting Na+/H+ exchanges and creating acidic positions during macropinocytosis (16). Our results implied that the endocytosis pathways were instrumental in electric field-mediated bleomycin uptake in the MCF-7 cells.

Non-permeabilizing and permeabilizing pulsed electric fields can both induce endocytosis. It has been reported that plasmid DNA (pDNA) is internalized by cells through an endocytosis-like process when used for electrotransfection (22-24). Antov et al. (3, 25) reported that a low unipolar pulse electric field with a 2.5-20 V/cm amplitude, 500 Hz frequency and exposure time of 1-10 minutes increased the uptake of macromolecules of dextran-FITC and BSA-FITC into the cells by stimulation of fluid-phase endocytosis. In another study, fluorescent-labelled macromolecules (dextran-FITC and β-galactosidase) were incorporated into membrane vesicles and cells by low pulsed electric fields that had intensities of 60 and 100 V/cm, 90 µs pulse duration, 1000 Hz and exposure times of 10 minutes. They reported that the underlying mechanism of this electric field-mediated uptake was an endocytic-like process (26). Mahrour et al. (27) observed that the electrical component of an electromagnetic field with an intensity of 1.2-8 V/cm, pulse duration of 75-580 µs, total exposure time of 5-90 minutes and frequency of 50-400 Hz was responsible for increased uptake of Lucifer yellow (LY) and FITC-dextran into cells by an endocytosis pathway. 

Our results revealed that LVHF could induce different endocytosis pathways, depending on the field conditions, and facilitate the entry of bleomycin, as an impermeable chemotherapy drug, into MCF-7 cells. For macropinocytosis, electric field strengths of 10 and 20 V/cm could stimulate this pathway of endocytosis. Clathrin-mediated endocytosis was induced at stronger field intensities (10, 30, 60 and 70 V/cm), while induction of the caveolae-mediated endocytosis occurred only at the lowest intensity of the electric field (10 V/cm). Caveolae-mediated endocytosis has the smallest vesicle diameter (60-80 nm) and occurs when the ligand binds with the receptor in the lipid-raft sections of the cell membrane (28). Because caveolae-mediated endocytosis has a low vesicle diameter, a small area of the cell membrane requires stimulation and a stronger electric field may not be necessary. Unlike the caveolae-mediated endocytosis, macropinocytosis has the highest vesicle diameter (200-500 µm) (29). Our results showed that, in addition to 10 V/cm, the 20 V/cm intensity could induce macropinocytosis; however, an increase in electric field voltage does not induce macropinocytosis. Stimulation of fluid-phase endocytosis by a low intensity electric field (2-20 V/cm) has been reported (3, 25). Clathrin-mediated endocytosis is initiated upon binding of ligands to receptors. Our findings imply that greater electric field intensities can induce clathrin-mediated endocytosis. Chang et al. (30) have demonstrated that eight electric pulses at 400-600 V/cm and 1 Hz frequency enhanced pDNA uptake via binding of pDNA to the cell membrane and endocytosis of membrane-bound pDNA. They reported the clathrin-mediated endocytosis played an important role in electrotransfection (31). It has been reported that a 900 MHz modulated electromagnetic field increased intracellular delivery of LY by clathrin-coated vesicles (32). 

Induction of endocytosis by electric fields can be attributed to the change in the charge density of the cell membrane. The electrostatic repulsion of a charged lipid in the cytosolic leaflet can induce spontaneous curvature in the plasma membrane (33, 34). Hirama et al. (35, 36) have reported that rapid removal of cholesterol or an increase in the density of phosphatidylserine leads to enhanced headgroup repulsion and an intensification of spontaneous membrane curvature that facilitates endocytosis. Also, different lipid densities within two leaflets induce spontaneous curvature in the bilayer membranes (37-39). The electric field induces local electrophoresis of the charge membrane and causes local segregation of charged components (lipids and proteins) on the membrane surface. This asymmetric surface charge distribution can lead to curvature of the membrane (3). Molecular dynamics simulations can show that membrane potentials cause curvature of the bilayer membrane (40).

## Conclusion

The results of this study imply that LVHF induces different endocytosis pathways in MCF-7 cells, which leads to increased bleomycin uptake.
